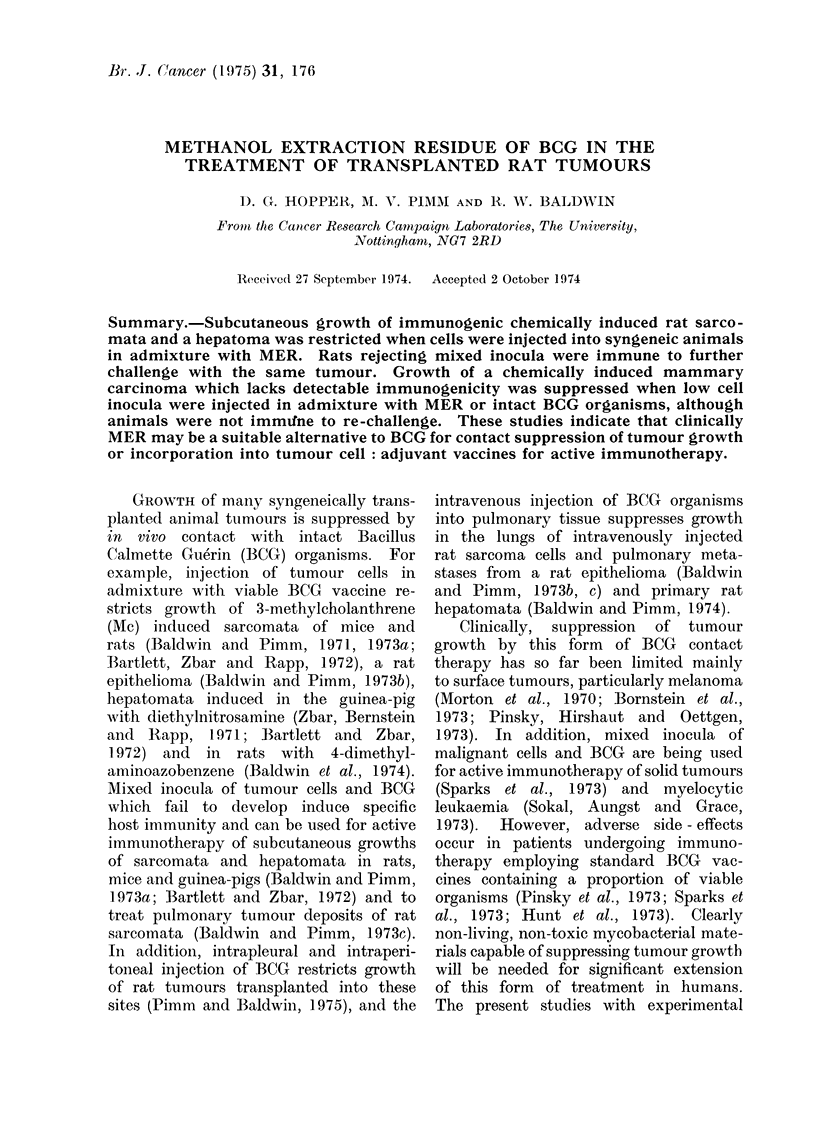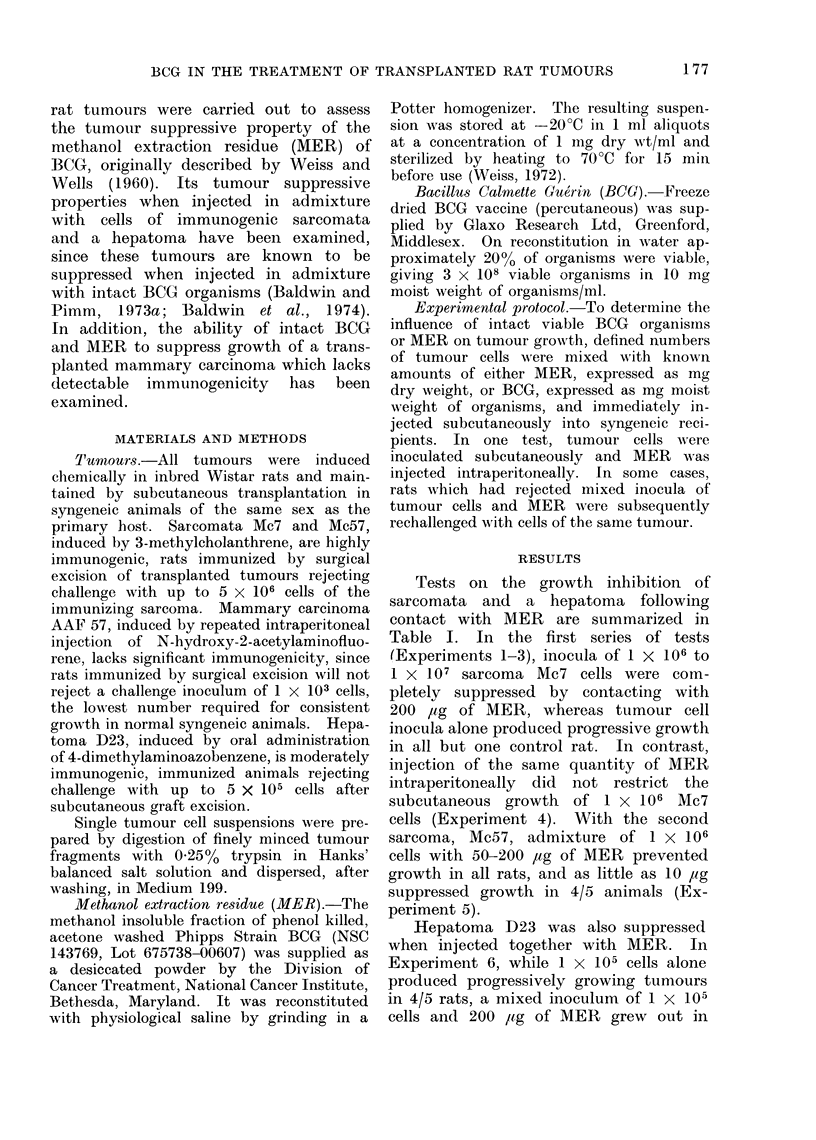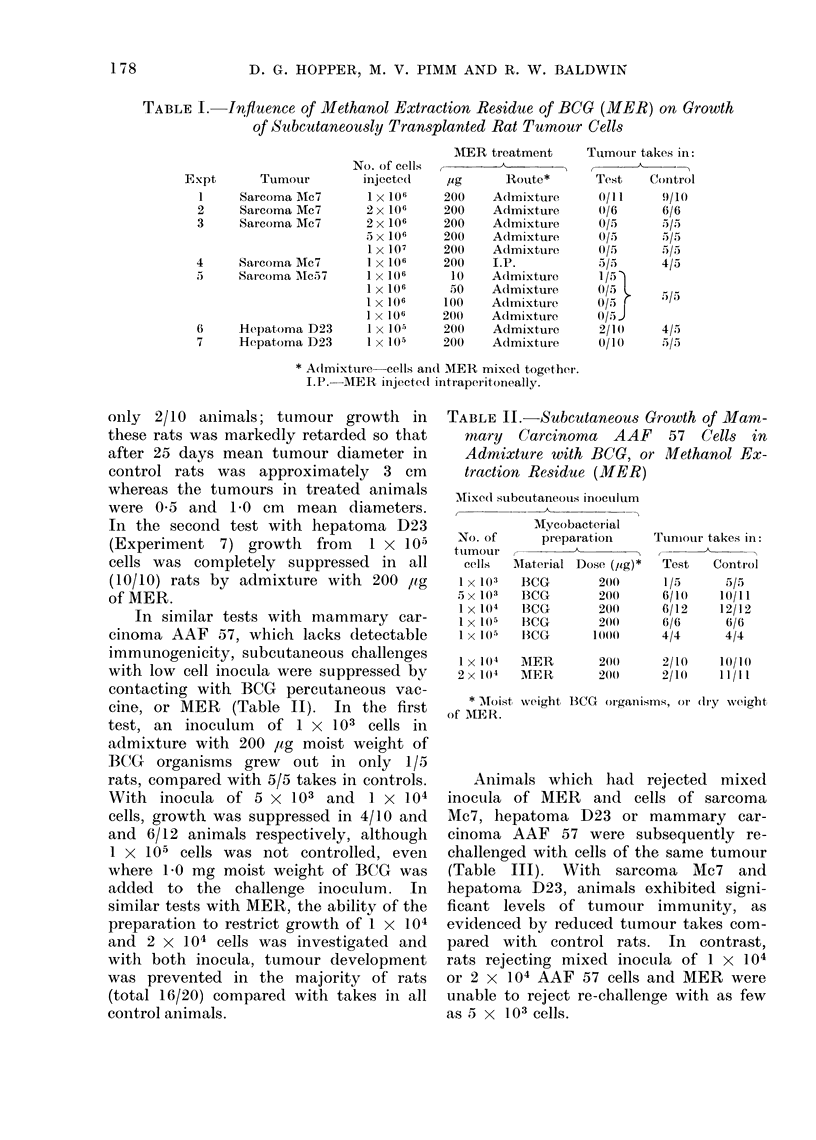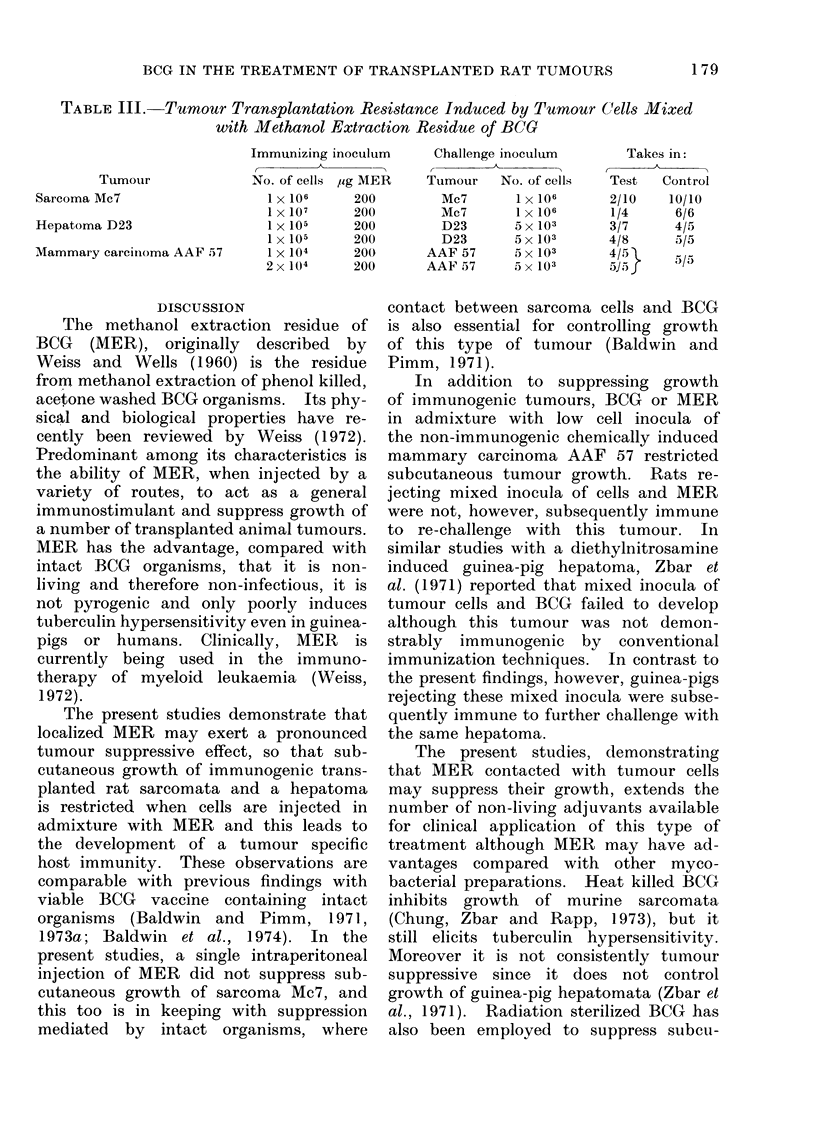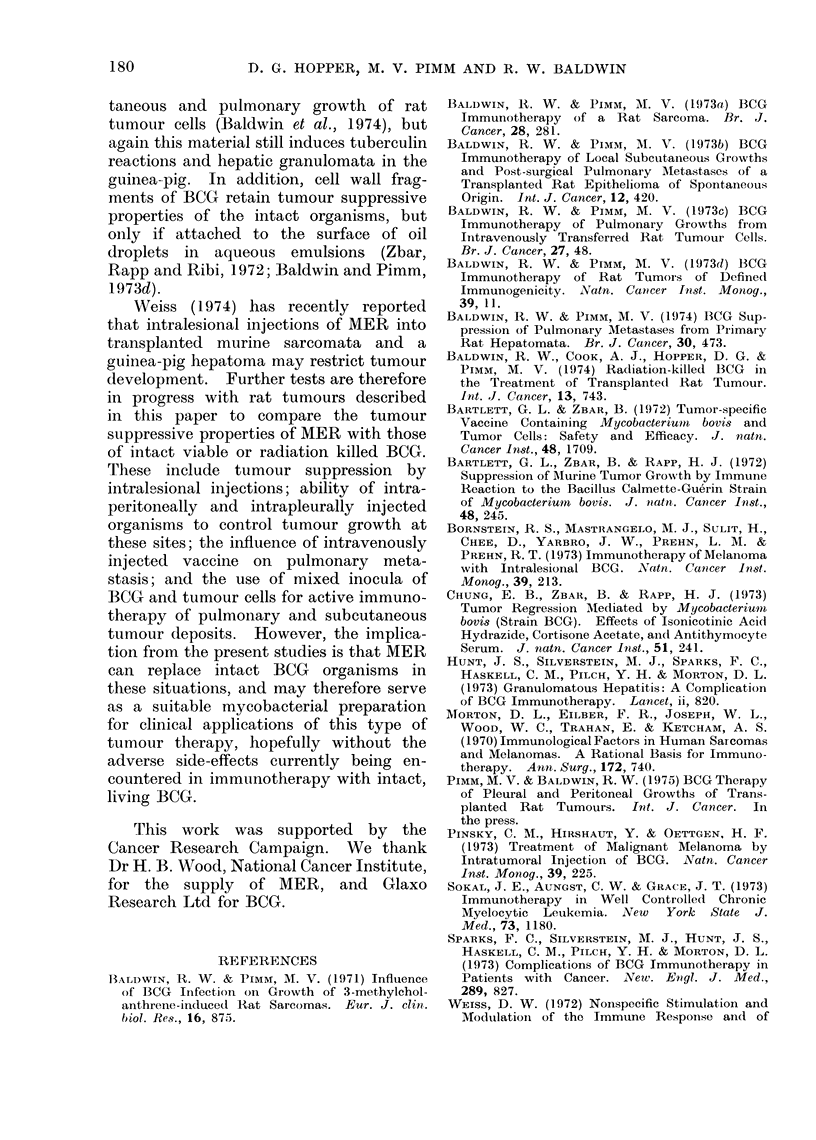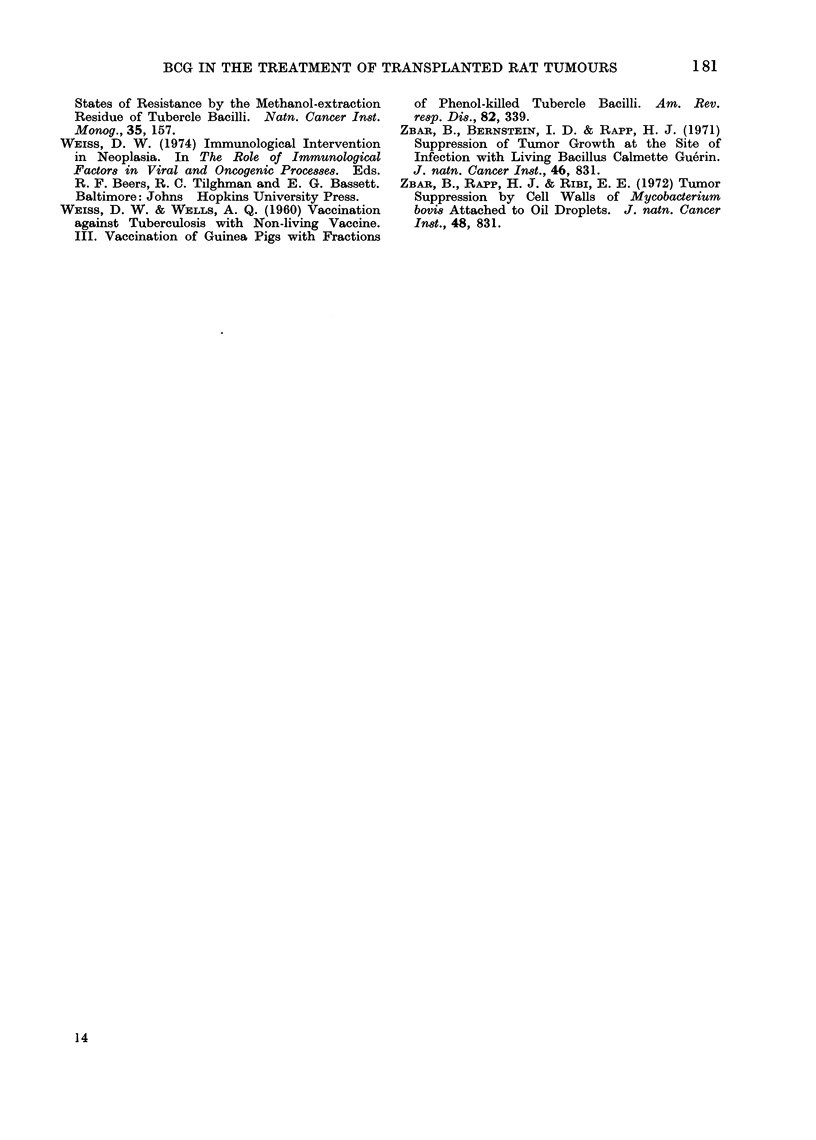# Methanol extraction residue of BCG in the treatment of transplanted rat tumours.

**DOI:** 10.1038/bjc.1975.23

**Published:** 1975-02

**Authors:** D. G. Hopper, M. V. Pimm, R. W. Baldwin

## Abstract

Subcutaneous growth of immunogenic chemically induced rat sarcomata and a hepatoma was restricted when cells were injected into syngeneic animals in admixture with MER. Rats rejecting mixed inocula were immune to further challenge with the same tumour. Growth of a chemically induced mammary carcinoma which lacks detectable immunogenicity was suppressed when low cell inocula were injected in admixture with MER or intact BCG organisms, although animals were not immune to re-challenge. These studies indicate that clinically MER may be a suitable alternative to BCG for contact suppression of tumour growth or incorporation into tumour cell:adjuvant vaccines for active immunotherapy.


Br. J. Cancer (1975) 31, 176

### METHANOL EXTRACTION RESIDUE OF BCG IN THE TREATMENT OF TRANSPLANTED RAT TUMOURS

D. G. HOPPER, M. V. PIMM AND R. W. BALDWIN

*From the Cancer Research Campaign Laboratories, The University, Nottingham, NG7 2RD*

Received 27 September 1974. Accepted 2 October 1974

**Summary.—Subcutaneous growth of immunogenic chemically induced rat sarcomata and a hepatoma was restricted when cells were injected into syngeneic animals in admixture with MER. Rats rejecting mixed inocula were immune to further challenge with the same tumour. Growth of a chemically induced mammary carcinoma which lacks detectable immunogenicity was suppressed when low cell inocula were injected in admixture with MER or intact BCG organisms, although animals were not immune to re-challenge. These studies indicate that clinically MER may be a suitable alternative to BCG for contact suppression of tumour growth or incorporation into tumour cell : adjuvant vaccines for active immunotherapy.**

GROWTH of many syngeneically transplanted animal tumours is suppressed by *in vivo* contact with intact Bacillus Calmette Guérin (BCG) organisms. For example, injection of tumour cells in admixture with viable BCG vaccine restricts growth of 3-methylcholanthrene (Mc) induced sarcomata of mice and rats (Baldwin and Pimm, 1971, 1973*a*; Bartlett, Zbar and Rapp, 1972), a rat epithelioma (Baldwin and Pimm, 1973*b*), hepatomata induced in the guinea-pig with diethylnitrosamine (Zbar, Bernstein and Rapp, 1971; Bartlett and Zbar, 1972) and in rats with 4-dimethylaminoazobenzene (Baldwin *et al.*, 1974). Mixed inocula of tumour cells and BCG which fail to develop induce specific host immunity and can be used for active immunotherapy of subcutaneous growths of sarcomata and hepatomata in rats, mice and guinea-pigs (Baldwin and Pimm, 1973*a*; Bartlett and Zbar, 1972) and to treat pulmonary tumour deposits of rat sarcomata (Baldwin and Pimm, 1973*c*). In addition, intrapleural and intraperitoneal injection of BCG restricts growth of rat tumours transplanted into these sites (Pimm and Baldwin, 1975), and the intravenous injection of BCG organisms into pulmonary tissue suppresses growth in the lungs of intravenously injected rat sarcoma cells and pulmonary metastases from a rat epithelioma (Baldwin and Pimm, 1973*b*, *c*) and primary rat hepatomata (Baldwin and Pimm, 1974).

Clinically, suppression of tumour growth by this form of BCG contact therapy has so far been limited mainly to surface tumours, particularly melanoma (Morton *et al.*, 1970; Bornstein *et al.*, 1973; Pinsky, Hirshaut and Oettgen, 1973). In addition, mixed inocula of malignant cells and BCG are being used for active immunotherapy of solid tumours (Sparks *et al.*, 1973) and myelocytic leukaemia (Sokal, Aungst and Grace, 1973). However, adverse side - effects occur in patients undergoing immunotherapy employing standard BCG vaccines containing a proportion of viable organisms (Pinsky *et al.*, 1973; Sparks *et al.*, 1973; Hunt *et al.*, 1973). Clearly non-living, non-toxic mycobacterial materials capable of suppressing tumour growth will be needed for significant extension of this form of treatment in humans. The present studies with experimental rat tumours were carried out to assess the tumour suppressive property of the methanol extraction residue (MER) of BCG, originally described by Weiss and Wells (1960). Its tumour suppressive properties when injected in admixture with cells of immunogenic sarcomata and a hepatoma have been examined, since these tumours are known to be suppressed when injected in admixture with intact BCG organisms (Baldwin and Pimm, 1973*a*; Baldwin *et al.*, 1974). In addition, the ability of intact BCG and MER to suppress growth of a transplanted mammary carcinoma which lacks detectable immunogenicity has been examined.

### MATERIALS AND METHODS

*Tumours.*—All tumours were induced chemically in inbred Wistar rats and maintained by subcutaneous transplantation in syngeneic animals of the same sex as the primary host. Sarcomata Mc7 and Mc57, induced by 3-methylcholanthrene, are highly immunogenic, rats immunized by surgical excision of transplanted tumours rejecting challenge with up to $5 \times 10^6$ cells of the immunizing sarcoma. Mammary carcinoma AAF 57, induced by repeated intraperitoneal injection of N-hydroxy-2-acetylaminofluorene, lacks significant immunogenicity, since rats immunized by surgical excision will not reject a challenge inoculum of $1 \times 10^3$ cells, the lowest number required for consistent growth in normal syngeneic animals. Hepatoma D23, induced by oral administration of 4-dimethylaminoazobenzene, is moderately immunogenic, immunized animals rejecting challenge with up to $5 \times 10^5$ cells after subcutaneous graft excision.

Single tumour cell suspensions were prepared by digestion of finely minced tumour fragments with 0·25% trypsin in Hanks' balanced salt solution and dispersed, after washing, in Medium 199.

*Methanol extraction residue* (*MER*).—The methanol insoluble fraction of phenol killed, acetone washed Phipps Strain BCG (NSC 143769, Lot 675738–00607) was supplied as a desiccated powder by the Division of Cancer Treatment, National Cancer Institute, Bethesda, Maryland. It was reconstituted with physiological saline by grinding in a Potter homogenizer. The resulting suspension was stored at −20°C in 1 ml aliquots at a concentration of 1 mg dry wt/ml and sterilized by heating to 70°C for 15 min before use (Weiss, 1972).

*Bacillus Calmette Guérin* (*BCG*).—Freeze dried BCG vaccine (percutaneous) was supplied by Glaxo Research Ltd, Greenford, Middlesex. On reconstitution in water approximately 20% of organisms were viable, giving $3 \times 10^8$ viable organisms in 10 mg moist weight of organisms/ml.

*Experimental protocol.*—To determine the influence of intact viable BCG organisms or MER on tumour growth, defined numbers of tumour cells were mixed with known amounts of either MER, expressed as mg dry weight, or BCG, expressed as mg moist weight of organisms, and immediately injected subcutaneously into syngeneic recipients. In one test, tumour cells were inoculated subcutaneously and MER was injected intraperitoneally. In some cases, rats which had rejected mixed inocula of tumour cells and MER were subsequently rechallenged with cells of the same tumour.

### RESULTS

Tests on the growth inhibition of sarcomata and a hepatoma following contact with MER are summarized in Table I. In the first series of tests (Experiments 1–3), inocula of $1 \times 10^6$ to $1 \times 10^7$ sarcoma Mc7 cells were completely suppressed by contacting with 200 $\mu$g of MER, whereas tumour cell inocula alone produced progressive growth in all but one control rat. In contrast, injection of the same quantity of MER intraperitoneally did not restrict the subcutaneous growth of $1 \times 10^6$ Mc7 cells (Experiment 4). With the second sarcoma, Mc57, admixture of $1 \times 10^6$ cells with 50–200 $\mu$g of MER prevented growth in all rats, and as little as 10 $\mu$g suppressed growth in 4/5 animals (Experiment 5).

Hepatoma D23 was also suppressed when injected together with MER. In Experiment 6, while $1 \times 10^5$ cells alone produced progressively growing tumours in 4/5 rats, a mixed inoculum of $1 \times 10^5$ cells and 200 $\mu$g of MER grew out in

TABLE I.—*Influence of Methanol Extraction Residue of BCG (MER) on Growth of Subcutaneously Transplanted Rat Tumour Cells*

| | | | MER treatment | | Tumour takes in: | |
|---|---|---|---|---|---|---|
| Expt | Tumour | No. of cells injected | μg | Route* | Test | Control |
| 1 | Sarcoma Mc7 | $1 \times 10^6$ | 200 | Admixture | 0/11 | 9/10 |
| 2 | Sarcoma Mc7 | $2 \times 10^6$ | 200 | Admixture | 0/6 | 6/6 |
| 3 | Sarcoma Mc7 | $2 \times 10^6$ | 200 | Admixture | 0/5 | 5/5 |
| | | $5 \times 10^6$ | 200 | Admixture | 0/5 | 5/5 |
| | | $1 \times 10^7$ | 200 | Admixture | 0/5 | 5/5 |
| 4 | Sarcoma Mc7 | $1 \times 10^6$ | 200 | I.P. | 5/5 | 4/5 |
| 5 | Sarcoma Mc57 | $1 \times 10^6$ | 10 | Admixture | 1/5 | 5/5 |
| | | $1 \times 10^6$ | 50 | Admixture | 0/5 | |
| | | $1 \times 10^6$ | 100 | Admixture | 0/5 | |
| | | $1 \times 10^6$ | 200 | Admixture | 0/5 | |
| 6 | Hepatoma D23 | $1 \times 10^5$ | 200 | Admixture | 2/10 | 4/5 |
| 7 | Hepatoma D23 | $1 \times 10^5$ | 200 | Admixture | 0/10 | 5/5 |

* Admixture—cells and MER mixed together.

I.P.—MER injected intraperitoneally.

only 2/10 animals; tumour growth in these rats was markedly retarded so that after 25 days mean tumour diameter in control rats was approximately 3 cm whereas the tumours in treated animals were 0·5 and 1·0 cm mean diameters. In the second test with hepatoma D23 (Experiment 7) growth from $1 \times 10^5$ cells was completely suppressed in all (10/10) rats by admixture with 200 μg of MER.

In similar tests with mammary carcinoma AAF 57, which lacks detectable immunogenicity, subcutaneous challenges with low cell inocula were suppressed by contacting with BCG percutaneous vaccine, or MER (Table II). In the first test, an inoculum of $1 \times 10^3$ cells in admixture with 200 μg moist weight of BCG organisms grew out in only 1/5 rats, compared with 5/5 takes in controls. With inocula of $5 \times 10^3$ and $1 \times 10^4$ cells, growth was suppressed in 4/10 and and 6/12 animals respectively, although $1 \times 10^5$ cells was not controlled, even where 1·0 mg moist weight of BCG was added to the challenge inoculum. In similar tests with MER, the ability of the preparation to restrict growth of $1 \times 10^4$ and $2 \times 10^4$ cells was investigated and with both inocula, tumour development was prevented in the majority of rats (total 16/20) compared with takes in all control animals.

TABLE II.—*Subcutaneous Growth of Mammary Carcinoma AAF 57 Cells in Admixture with BCG, or Methanol Extraction Residue (MER)*

| Mixed subcutaneous inoculum | | | | |
|---|---|---|---|---|
| No. of tumour cells | Mycobacterial preparation | | Tumour takes in: | |
| | Material | Dose (μg)* | Test | Control |
| $1 \times 10^3$ | BCG | 200 | 1/5 | 5/5 |
| $5 \times 10^3$ | BCG | 200 | 6/10 | 10/11 |
| $1 \times 10^4$ | BCG | 200 | 6/12 | 12/12 |
| $1 \times 10^5$ | BCG | 200 | 6/6 | 6/6 |
| $1 \times 10^5$ | BCG | 1000 | 4/4 | 4/4 |
| $1 \times 10^4$ | MER | 200 | 2/10 | 10/10 |
| $2 \times 10^4$ | MER | 200 | 2/10 | 11/11 |

* Moist weight BCG organisms, or dry weight of MER.

Animals which had rejected mixed inocula of MER and cells of sarcoma Mc7, hepatoma D23 or mammary carcinoma AAF 57 were subsequently rechallenged with cells of the same tumour (Table III). With sarcoma Mc7 and hepatoma D23, animals exhibited significant levels of tumour immunity, as evidenced by reduced tumour takes compared with control rats. In contrast, rats rejecting mixed inocula of $1 \times 10^4$ or $2 \times 10^4$ AAF 57 cells and MER were unable to reject re-challenge with as few as $5 \times 10^3$ cells.

TABLE III.—*Tumour Transplantation Resistance Induced by Tumour Cells Mixed with Methanol Extraction Residue of BCG*

| | Immunizing inoculum | | Challenge inoculum | | Takes in: | |
|---|---|---|---|---|---|---|
| Tumour | No. of cells | μg MER | Tumour | No. of cells | Test | Control |
| Sarcoma Mc7 | $1 \times 10^6$ | 200 | Mc7 | $1 \times 10^6$ | 2/10 | 10/10 |
| | $1 \times 10^7$ | 200 | Mc7 | $1 \times 10^6$ | 1/4 | 6/6 |
| Hepatoma D23 | $1 \times 10^5$ | 200 | D23 | $5 \times 10^3$ | 3/7 | 4/5 |
| | $1 \times 10^5$ | 200 | D23 | $5 \times 10^3$ | 4/8 | 5/5 |
| Mammary carcinoma AAF 57 | $1 \times 10^4$ | 200 | AAF 57 | $5 \times 10^3$ | 4/5 | 5/5 |
| | $2 \times 10^4$ | 200 | AAF 57 | $5 \times 10^3$ | 5/5 | |

### DISCUSSION

The methanol extraction residue of BCG (MER), originally described by Weiss and Wells (1960) is the residue from methanol extraction of phenol killed, acetone washed BCG organisms. Its physical and biological properties have recently been reviewed by Weiss (1972). Predominant among its characteristics is the ability of MER, when injected by a variety of routes, to act as a general immunostimulant and suppress growth of a number of transplanted animal tumours. MER has the advantage, compared with intact BCG organisms, that it is non-living and therefore non-infectious, it is not pyrogenic and only poorly induces tuberculin hypersensitivity even in guinea-pigs or humans. Clinically, MER is currently being used in the immunotherapy of myeloid leukaemia (Weiss, 1972).

The present studies demonstrate that localized MER may exert a pronounced tumour suppressive effect, so that subcutaneous growth of immunogenic transplanted rat sarcomata and a hepatoma is restricted when cells are injected in admixture with MER and this leads to the development of a tumour specific host immunity. These observations are comparable with previous findings with viable BCG vaccine containing intact organisms (Baldwin and Pimm, 1971, 1973*a*; Baldwin *et al.*, 1974). In the present studies, a single intraperitoneal injection of MER did not suppress subcutaneous growth of sarcoma Mc7, and this too is in keeping with suppression mediated by intact organisms, where contact between sarcoma cells and BCG is also essential for controlling growth of this type of tumour (Baldwin and Pimm, 1971).

In addition to suppressing growth of immunogenic tumours, BCG or MER in admixture with low cell inocula of the non-immunogenic chemically induced mammary carcinoma AAF 57 restricted subcutaneous tumour growth. Rats rejecting mixed inocula of cells and MER were not, however, subsequently immune to re-challenge with this tumour. In similar studies with a diethylnitrosamine induced guinea-pig hepatoma, Zbar *et al.* (1971) reported that mixed inocula of tumour cells and BCG failed to develop although this tumour was not demonstrably immunogenic by conventional immunization techniques. In contrast to the present findings, however, guinea-pigs rejecting these mixed inocula were subsequently immune to further challenge with the same hepatoma.

The present studies, demonstrating that MER contacted with tumour cells may suppress their growth, extends the number of non-living adjuvants available for clinical application of this type of treatment although MER may have advantages compared with other mycobacterial preparations. Heat killed BCG inhibits growth of murine sarcomata (Chung, Zbar and Rapp, 1973), but it still elicits tuberculin hypersensitivity. Moreover it is not consistently tumour suppressive since it does not control growth of guinea-pig hepatomata (Zbar *et al.*, 1971). Radiation sterilized BCG has also been employed to suppress subcutaneous and pulmonary growth of rat tumour cells (Baldwin *et al.*, 1974), but again this material still induces tuberculin reactions and hepatic granulomata in the guinea-pig. In addition, cell wall fragments of BCG retain tumour suppressive properties of the intact organisms, but only if attached to the surface of oil droplets in aqueous emulsions (Zbar, Rapp and Ribi, 1972; Baldwin and Pimm, 1973*d*).

Weiss (1974) has recently reported that intralesional injections of MER into transplanted murine sarcomata and a guinea-pig hepatoma may restrict tumour development. Further tests are therefore in progress with rat tumours described in this paper to compare the tumour suppressive properties of MER with those of intact viable or radiation killed BCG. These include tumour suppression by intralesional injections; ability of intraperitoneally and intrapleurally injected organisms to control tumour growth at these sites; the influence of intravenously injected vaccine on pulmonary metastasis; and the use of mixed inocula of BCG and tumour cells for active immunotherapy of pulmonary and subcutaneous tumour deposits. However, the implication from the present studies is that MER can replace intact BCG organisms in these situations, and may therefore serve as a suitable mycobacterial preparation for clinical applications of this type of tumour therapy, hopefully without the adverse side-effects currently being encountered in immunotherapy with intact, living BCG.

This work was supported by the Cancer Research Campaign. We thank Dr H. B. Wood, National Cancer Institute, for the supply of MER, and Glaxo Research Ltd for BCG.

### REFERENCES

BALDWIN, R. W. & PIMM, M. V. (1971) Influence of BCG Infection on Growth of 3-methylcholanthrene-induced Rat Sarcomas. *Eur. J. clin. biol. Res.*, **16**, 875.

BALDWIN, R. W. & PIMM, M. V. (1973*a*) BCG Immunotherapy of a Rat Sarcoma. *Br. J. Cancer*, **28**, 281.

BALDWIN, R. W. & PIMM, M. V. (1973*b*) BCG Immunotherapy of Local Subcutaneous Growths and Post-surgical Pulmonary Metastases of a Transplanted Rat Epithelioma of Spontaneous Origin. *Int. J. Cancer*, **12**, 420.

BALDWIN, R. W. & PIMM, M. V. (1973*c*) BCG Immunotherapy of Pulmonary Growths from Intravenously Transferred Rat Tumour Cells. *Br. J. Cancer*, **27**, 48.

BALDWIN, R. W. & PIMM, M. V. (1973*d*) BCG Immunotherapy of Rat Tumors of Defined Immunogenicity. *Natn. Cancer Inst. Monog.*, **39**, 11.

BALDWIN, R. W. & PIMM, M. V. (1974) BCG Suppression of Pulmonary Metastases from Primary Rat Hepatomata. *Br. J. Cancer*, **30**, 473.

BALDWIN, R. W., COOK, A. J., HOPPER, D. G. & PIMM, M. V. (1974) Radiation-killed BCG in the Treatment of Transplanted Rat Tumour. *Int. J. Cancer*, **13**, 743.

BARTLETT, G. L. & ZBAR, B. (1972) Tumor-specific Vaccine Containing *Mycobacterium bovis* and Tumor Cells: Safety and Efficacy. *J. natn. Cancer Inst.*, **48**, 1709.

BARTLETT, G. L., ZBAR, B. & RAPP, H. J. (1972) Suppression of Murine Tumor Growth by Immune Reaction to the Bacillus Calmette-Guérin Strain of *Mycobacterium bovis*. *J. natn. Cancer Inst.*, **48**, 245.

BORNSTEIN, R. S., MASTRANGELO, M. J., SULIT, H., CHEE, D., YARBRO, J. W., PREHN, L. M. & PREHN, R. T. (1973) Immunotherapy of Melanoma with Intralesional BCG. *Natn. Cancer Inst. Monog.*, **39**, 213.

CHUNG, E. B., ZBAR, B. & RAPP, H. J. (1973) Tumor Regression Mediated by *Mycobacterium bovis* (Strain BCG). Effects of Isonicotinic Acid Hydrazide, Cortisone Acetate, and Antithymocyte Serum. *J. natn. Cancer Inst.*, **51**, 241.

HUNT, J. S., SILVERSTEIN, M. J., SPARKS, F. C., HASKELL, C. M., PILCH, Y. H. & MORTON, D. L. (1973) Granulomatous Hepatitis: A Complication of BCG Immunotherapy. *Lancet*, ii, 820.

MORTON, D. L., EILBER, F. R., JOSEPH, W. L., WOOD, W. C., TRAHAN, E. & KETCHAM, A. S. (1970) Immunological Factors in Human Sarcomas and Melanomas. A Rational Basis for Immunotherapy. *Ann. Surg.*, **172**, 740.

PIMM, M. V. & BALDWIN, R. W. (1975) BCG Therapy of Pleural and Peritoneal Growths of Transplanted Rat Tumours. *Int. J. Cancer.* In the press.

PINSKY, C. M., HIRSHAUT, Y. & OETTGEN, H. F. (1973) Treatment of Malignant Melanoma by Intratumoral Injection of BCG. *Natn. Cancer Inst. Monog.*, **39**, 225.

SOKAL, J. E., AUNGST, C. W. & GRACE, J. T. (1973) Immunotherapy in Well Controlled Chronic Myelocytic Leukemia. *New York State J. Med.*, **73**, 1180.

SPARKS, F. C., SILVERSTEIN, M. J., HUNT, J. S., HASKELL, C. M., PILCH, Y. H. & MORTON, D. L. (1973) Complications of BCG Immunotherapy in Patients with Cancer. *New. Engl. J. Med.*, **289**, 827.

WEISS, D. W. (1972) Nonspecific Stimulation and Modulation of the Immune Response and of

States of Resistance by the Methanol-extraction Residue of Tubercle Bacilli. *Natn. Cancer Inst. Monog.*, **35**, 157.

WEISS, D. W. (1974) Immunological Intervention in Neoplasia. In *The Role of Immunological Factors in Viral and Oncogenic Processes*. Eds. R. F. Beers, R. C. Tilghman and E. G. Bassett. Baltimore: Johns Hopkins University Press.

WEISS, D. W. & WELLS, A. Q. (1960) Vaccination against Tuberculosis with Non-living Vaccine. III. Vaccination of Guinea Pigs with Fractions of Phenol-killed Tubercle Bacilli. *Am. Rev. resp. Dis.*, **82**, 339.

ZBAR, B., BERNSTEIN, I. D. & RAPP, H. J. (1971) Suppression of Tumor Growth at the Site of Infection with Living Bacillus Calmette Guérin. *J. natn. Cancer Inst.*, **46**, 831.

ZBAR, B., RAPP, H. J. & RIBI, E. E. (1972) Tumor Suppression by Cell Walls of *Mycobacterium bovis* Attached to Oil Droplets. *J. natn. Cancer Inst.*, **48**, 831.